# Evaluation of a New Method to Track Changes in Vision at Home for Children Undergoing Amblyopia Treatment

**DOI:** 10.22599/bioj.172

**Published:** 2021-04-09

**Authors:** Anna O’Connor, Martha Waters, Laura England, Ashli Milling, Hazel Kay

**Affiliations:** 1 University of Liverpool, GB; 2 Manchester Royal Eye Hospital, GB; 3 Kay Pictures, GB

**Keywords:** amblyopia, visual acuity, test retest variability, optotype, app

## Abstract

**Purpose:**

A new amblyopia tracker app has been designed to provide parents with a method of monitoring a child’s vision by presenting a single optotype size that the tester moves to identify the furthest distance the optotypes can be seen. The aim of this study is to evaluate this methodology in adults, comparing the findings to visual acuity (VA) measured with the iSight app and to determine the test retest variability.

**Methods:**

Adults, aged 18–39 years, with no known eye condition and VA ≤ 0.7 logMAR were recruited. Bangerter filters were used to simulate amblyopia, where VA was reduced below 0.0 with an interocular difference of at least 0.2 logMAR. Testing for both apps was performed monocularly, with the test order being randomised.

**Results:**

Data from 32 subjects were analysed. For the test retest variability analysis, paired t-tests showed no statistically significant difference between the tests for either eye, either app or the interocular acuity difference (p > 0.3 in all cases). Bland Altman plots showed similar limits of agreement between the two apps. When comparing measurements between the apps there was no statistically significant difference on the first or second test, either eye or the interocular acuity difference (p > 0.5 in all cases).

**Conclusion:**

The results support the theory that changing distance is a valid method of assessing VA as the measurements agree well with the standard approach of reducing optotype size. Test retest variability is similar between the two apps and there is good agreement between the measurements.

## Introduction

Amblyopia affects the eyesight of 2–3% of children in the UK and Europe (Fu et al. 2020; Hashemi et al. 2018), requiring home treatment with occlusion or atropine and careful, regular monitoring of the child’s visual acuity (VA). Children undergoing amblyopia treatment require serial measurements of their VA to monitor any changes in response to treatment, which means that patients must attend for regular eye clinic appointments to monitor changes. During the COVID-19 pandemic these regular hospital appointments were postponed for many months, with guidance from the British and Irish Orthoptic Society to reduce occlusion time due to the lack of formal remote monitoring tools.

There are numerous apps available to assess VA, which are widely accessible, with some orthoptists recommending the use of apps for remote testing during the pandemic (Rowe et al. 2020). But the current guidance from the Royal College of Ophthalmologists in relation to paediatric ophthalmology (published 18 November 2020) states that ‘visual acuity apps do exist …, but they are not fully validated against standard visual acuity tests’ (Royal College of Ophthalmologists 2020). The American Academy of Ophthalmology guidance states that ‘online vision testing should be limited to healthy adults (ages 18–39 years)’ (American Academy of Ophthalmology 2020). This guidance is based on the lack of evidence in relation to assessing VA in children. VA measurements using letter-based apps have demonstrated good agreement with standard clinical tests (Ansell et al. 2020; Bastawrous et al. 2015). There are apps to assess VA in children, but they are designed for use as a screening device used by trained clinicians using the tumbling E optotype (Di Foggia et al. 2020; Zhao et al. 2019). Recent reviews have identified a lack of validation of tests specifically for home use for virtual consultation telemedicine (Nagra et al. 2020; Steren et al. 2021).

The Amblyopia Tracker app (Kay Pictures Ltd, Tring, UK) has been developed as a way for parents to monitor changes in a child’s vision at home while they are being treated for amblyopia using letter or picture optotypes, rather than providing a measure of visual acuity. The intention being that it is provided to parents as a monitoring tool to give clinicians and parents useful feedback on their child’s amblyopia progress between clinical appointments. It aims to minimise the issues associated with the home use of professional eye charts by measuring and recording any changes in vision in a different way, so that parents can see vision changes and improvements in a simple chart, rather than looking for direct VA comparisons to clinical measures that may not be fully understood. To perform the test, parents hold the phone and follow the instructions given on the phone, so the child only needs to respond to what they see. Results are provided as the furthest distance the optotype can be seen, and repeated tests are summarised in chart format to allow parents to see any changes over time. Also, the app will base the starting point of the test on the previous measurement to reduce the time to complete it.

Using an app regularly at home to monitor changes in vision in children with amblyopia has the potential to allow the eye professional treating patients to, when appropriate, safely lengthen the time interval between hospital appointments by reviewing the app results. This is important to avoid vision loss during the immediate COVID-19 pandemic and has the ongoing potential for significant National Health Service (NHS) savings from fewer hospital appointments being required. In addition, it is known that parent and patient understanding of their condition and interaction with progress improves compliance with amblyopia treatment (Vagge & Nelson 2017). Providing parents a way to monitor changes and possible improvements in their child’s visual condition in a chart could have a positive effect on compliance with treatment.

The Amblyopia Tracker app uses the principle of calculating VA based on the angle subtended at the eye with changing distance, rather than changing optotype size. Theoretically, there should be no difference between measurements taken by changing the optotype size or changing the test distance with a known size of optotype, but no evidence was found to validate this alternative method of vision assessment. There are, however, a few studies that have measured vision at different distances with a standard chart. One report found no significant difference in visual acuity measurements taken at 3 or 6 metres (Geddes et al. 1966). Four studies suggest that test distances at less than 2 metres tend to underestimate visual acuity; however, all these studies were on adults with age-related macular degeneration who had significantly reduced vision (Dong et al. 2002). Therefore, there is a need for validation that the theory works in practise and the test process is repeatable and reliable.

This study is the first phase in the evaluation of the Amblyopia Tracker app. As the app is designed to monitor changes in vision, the most important measure of accuracy is the test-retest variability; therefore, this is the primary outcome of this study. In addition, variability results are compared with results from the iSight app (Kay Pictures Ltd, Tring, UK) on adults. Testing was completed with Bangerter filters to simulate amblyopia and repeated to enable the test-retest variability to be calculated.

## Methods

Data were collected at Manchester Royal Eye Hospital during a two-week period in October 2020. The study conformed to the principles of the Declaration of Helsinki, and ethical approval was obtained from the NHS research ethics committee, with all participants providing informed consent.

Inclusion criteria were subject age 18–39 years, working at the hospital, with no known eye condition, wearing their habitual correction. As there was a screen size limit of the iPhone used for the iSight Pro app, VA had to be better than or equal to 0.7 logMAR. Adults were tested to avoid the impact of cognitive ability on the results, and the age limits were used to prevent the potential impact of presbyopia when bringing the phone closer to the subject. The same researcher, smartphone (iPhone 6), room, and lighting conditions were used to ensure standardisation. There were three phases to the data collection process: initial vision testing, screening phase to determine the appropriate Bangerter filter to sufficiently reduce VA, and the testing phase.

## Vision testing

Both the standard visual acuity test (using Kay iSight Pro) and the Amblyopia Tracker were presented on an iPhone using a single ETDRS letter optotype in an optimal crowding box. For the tracker measurement, a fixed optotype size is presented at various test distances governed by a simple staircase in the app and a score system where three out of five correct is a pass at any size. The test requires users to measure their test distances to a maximum of 300 centimetres, which equates to a visual acuity score of 0.0 logMAR. The iSight app used the same six ETDRS letters, which matched the tracker app optotypes.

The fixed optotype size for the tracker app was chosen as the most appropriate to be used on any smartphone without being too small for accurate, clear display. However, this size does limit the best achievable score to 0.0 logMAR at 300 cm. As many participants had VA better than 0.0, Bangerter filters were introduced to bring acuities within the app’s range and to simulate amblyopia. In addition to the comparison test, test-retest variability was determined for both test processes by repeated testing of each eye with each test. All testing was completed within one session.

### Initial vision testing and screening phase

An initial monocular threshold vision with habitual correction was performed using the iSight app to inform whether Bangerter filters were required for the testing phase and to guide which filters to start with. While the Amblyopia Tracker app distances equate to VA from 0.0 to 1.3 logMAR, the iPhone could only display a maximum of 0.7 logMAR; therefore, vision had to be within a testing range of 0.0 and 0.7 logMAR. Therefore, the screening phase was used to ensure vision was within the appropriate range to enable recording on both apps using Bangerter filters. This was not a complete threshold measure as it was simply a basis to ensure the appropriate filters were selected to simulate amblyopia. An interocular acuity difference of at least two logMAR lines was required. When appropriate, this was achieved with a Bangerter filter bar using filters ranging between <0.1 and 1.0 to blur each participant’s vision appropriately. The participant was asked to hold the bar with a filter over one eye while the other eye was occluded. The iSight pro app displayed one crowded letter at each logMAR acuity size, descending from 0.7 logMAR, the line with the last correct letter was recorded as the screening vision.

Some participants achieved vision within the required range in screening but then went on to achieve better than 0.0 logMAR in the testing phase. As <0.0 logMAR is outside the testing range of the Tracker app, the decision was made to ensure the filters blurred participants to at least 0.2 logMAR in the better eye in the screening phase to account for any improvement in vision during the testing phase.

### Testing Phase

Monocular vision was tested with the iSight Pro and Amblyopia Tracker app. The order of testing was randomised along with which eye was tested first. Each app test was repeated to assess test-retest variability. Each participant had their vision tested a total of four times in each eye.

## Process for iSight Pro app

The participant occluded the eye not being tested and was instructed to hold the chosen Bangerter filter in front of the eye being tested. Each participant was instructed to read aloud each letter displayed on the screen and to inform the investigators if they were unable to see it. Participants’ threshold visual acuity was assessed with their habitual correction. VA was measured monocularly, and the app was set to display one single-crowded letter at a time. The following protocol was used to determine the threshold VA:

1. Starting at 0.7 logMAR, test one letter per line until participant gets one wrong;
2. When one letter was incorrectly identified, return to the line above and test 4 more letters on this line;
3. If at least 3/5 correct, continue to next size down again;
4. Test 5 letters on each line until less than 3/5 correctly identified;
5. If less than 3/5 correct, stop testing and record VA based on the number of letters that were correctly identified on this line;
6. If 0/5 correct, record VA based on the number of letters that were correctly identified on the line before.

## Process for the Amblyopia Tracker app

The participant occluded the eye not being tested and was instructed to hold the chosen Bangerter filter over the eye being tested. A 3 m tape measure was fixed to the floor, beginning beneath the participant’s seat.

1. App displays the distance from the participant that the tester should hold the phone, (range 15–300 cm) starting at 30 cm distance.
2. Investigator taps ‘ready’ to display the first letter.
3. Investigator taps ‘correct’ or ‘incorrect’ depending on the participant’s answer.
4. Each participant is tested with up to five letters at any one distance, as specified by the app.
5. Depending on whether three out of five letters are correctly identified or not, the app instructs the researcher to move to a new, specified distance along the logarithmic scale between 15 cm and 300 cm. Step sizes are automated with a three up, one down process employed to identify the approximate distance at which the optotype is seen, followed by single step sizes to determine the threshold.
6. Repeat steps 1–4 until the app displays the final distance score to be recorded.

Fixed step sizes were calculated following a staircase to minimise the number of steps needed to reach the final distance measurement. If the first optotype was correctly identified, the distance was increased by three step sizes (one step size equating to one logMAR line change); this continued until an incorrect response was given. Then the tester was instructed to bring the phone forward one step size at a time until the subject could identify three out of five optotypes correctly.

## Statistical analysis

Tracker distances were converted to logMAR values for analysis. The iSight app VA scores were rounded to the nearest line (where a minimum of three out of five optotypes per line were correctly identified). Data were analysed using the Kolmogorov Smirnov test for normality. Only the second test of the left eye on the Amblyopia Tracker app was not normally distributed; therefore, parametric tests were used. To analyse the test-retest data, paired t-tests were used and Bland Altman plots were used to evaluate the agreement between tests. For the test-retest variability analysis, the mean bias was calculated as the first test VA minus the second test VA, and for the comparison between tests, the Amblyopia tracker app VA was subtracted from the iSight VA. Limits of agreement were calculated as 1.96 times the standard deviation of the difference between the two tests. The sample size was determined using the Altman nomogram. To detect a difference of 0.1 logMAR (chosen to reflect a clinically significant difference) and a standard deviation of 0.1 logMAR based on previously published data using the Kay pictures book (O’Connor & Milling 2020), a sample size of 30 was required.

## Results

Thirty-six adults participated, mean age 29 years (SD 5.4). Four were excluded from the analysis as their VA on the iSight app was better than 0.0 logMAR, despite VA worse than 0.0 logMAR during the screening phase, leaving 32 participants in the analysis.

### Test-retest variability

Baseline VA using the iSight app without the Bangerter filters was RE –0.088(±0.151) and LE –0.089(±0.170) logMAR. One participant required no filters as their VA was within the required limits for the tracker app; four others only required a filter for one eye.

**Figure 1:**
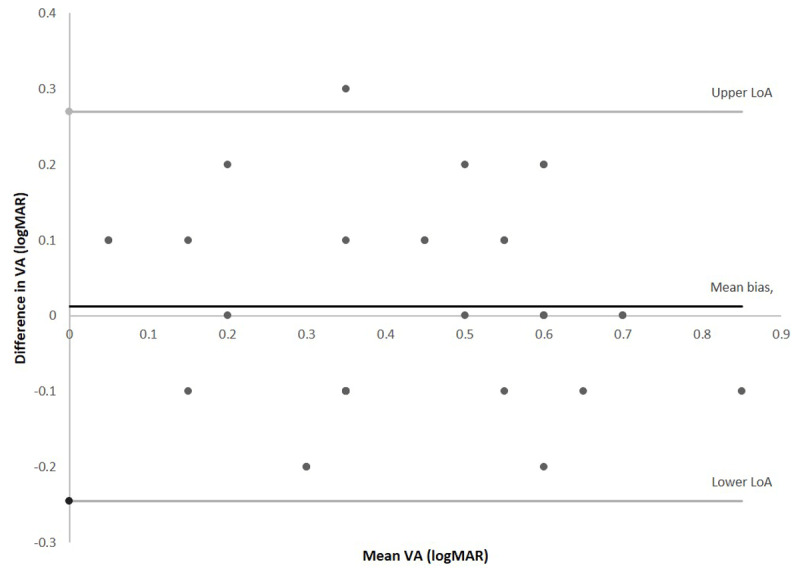
Bland Altman showing agreement between test one and test two of the right eye for the tracker app, comparing the mean of the two tests against the difference.

As shown in ***Table 1***, there is no statistically significant difference between test one and two for either measure of VA. The mean bias and limits of agreement were very similar across all tests, with a mean bias close to zero for both VA tests on first and second test. ***Figure 1*** is a Bland Altman plot showing the agreement between test one and two of the right eye for the Amblyopia Tracker app.

**Table 1:** VA data for each eye and each test from the iSight and Amblyopia Tracker apps, mean bias, and upper and lower limits of agreement (LoA).


	VA ISIGHT APP (LOGMAR) MEAN ± SD		TRACKER APP LOGMAR VALUE MEAN ± SD	

	RE	LE	RE	LE

**Test 1**	0.44 ± 0.204	0.38 ± 0.201	0.43 ± 0.214	0.39 ± 0.20

**Test 2**	0.42 ± 0.219	0.38 ± 0.214	0.44 ± 0.238	0.38 ± 0.23

**Paired t-test p value**	0.34	1.0	0.66	0.39

**Mean bias ± SD**	0.02 ± 0.126	0 ± 0.114	–0.01 ± 0.120	0.02 ± 0.120

**Upper LoA**	0.269	0.222	0.226	0.255

**Lower LoA**	–0.226	–0.222	–0.245	–0.217


**Figure 2:**
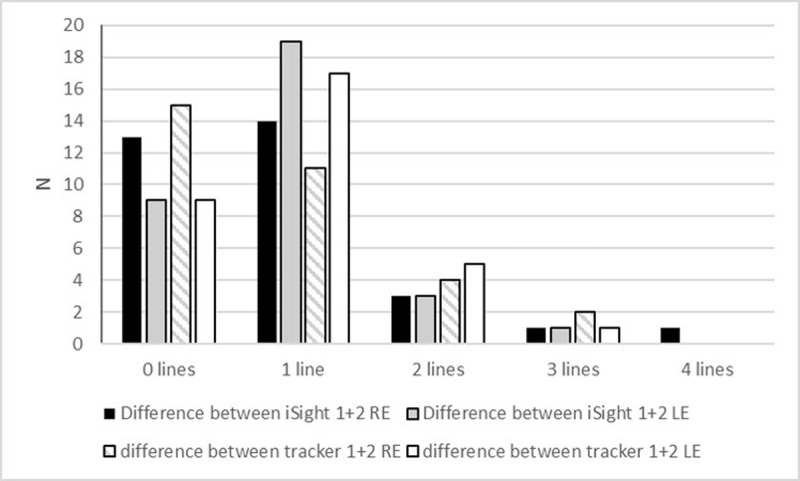
Bar chart showing the numbers of participants and the difference in VA between test one and two for each eye and each test.

As there are overlapping data points on the Bland Altman plot, ***Figure 2*** shows the numbers of subjects for each line of difference between the first and second test. The proportions of subjects with one or no lines difference between the first and second test on the iSight test was 84% for the right eye and 88% for the left eye. For the tracker app it was 81% for each eye.

As the aim of the tracker app is to monitor VA in patients with amblyopia, it is important to analyse the interocular acuity difference data in combination with the monocular VA scores. Paired t-test analysis showed no statistically significant difference in the IAD for either method on either test (iSight app p = 0.5, tracker app p = 0.4).

## Comparison between tests

**Figure 3:**
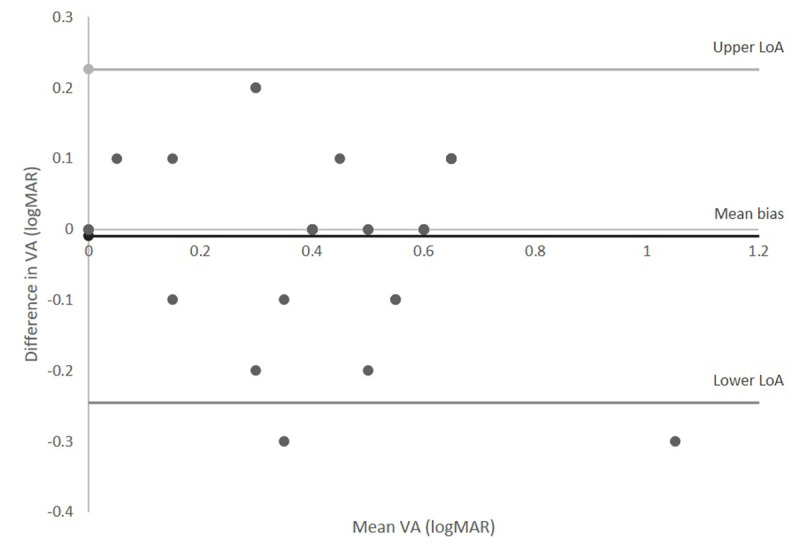
Bland Altman plot of the right eye data comparing responses between the two test methods.

***Table 2*** shows the VA results are very similar between tests with a mean bias close to zero, as shown in ***Figure 3***, and no statistically significant difference found.

**Table 2:** Comparison of the VA results between the two testing methods.


	VA ISIGHT APP (LOGMAR) – VA TRACKER APP			

	RE TEST 1	RE TEST 2	LE TEST 1	LE TEST 2

**Mean bias±SD**	0.013 ± 0.131	–0.019 ± 0.164	–0.009 ± 0.106	0.009 ± 0.112

**Upper LoA**	0.270	0.302	0.198	0.228

**Lower LoA**	–0.245	–0.339	–0.217	–0.210

**Paired t-test p value**	0.59	0.52	0.62	0.64


When comparing the IAD between the tests there was no statistically significant difference on test one or two (see ***Table 3***).

**Table 3:** Interocular acuity difference (mean ± SD) for the iSight app and tracker app.


ISIGHT IAD			TRACKER APP IAD		

TEST 1	TEST 2	DIFFERENCE	TEST ONE	TEST TWO	DIFFERENCE

0.056 ± 0.350	0.034 ± 0.378	0.022 ± 0.181	0.034 ± 0.357	0.063 ± 0.390	–0.028 ± 0.195


## Discussion

This first phase of evaluation of the new Amblyopia Tracker app was designed to determine if the concept of changing the test distance rather than optotype size produces equivalent VA responses to the standard test method. The results support the theory behind the change in approach to assessing VA and demonstrate that when clinicians use the tests on cooperative adult subjects, test-retest variability is equivalent to the iSight app and there is good agreement between the tests.

While there is no statistically significant difference when comparing test one and two and methods, there is some variability. A degree of variability is expected, with previous data showing that when assessing VA using the HOTV test, 93% (n = 163) of responses had either one line or no difference on retest (Holmes et al. 2001) and the same finding of 93% (n = 42) for Lea symbols (Chen et al. 2006). One app, designed for children with a C optotype presented in a game format at near, is reported to have limits of agreement of ±0.208 (Aslam et al. 2016). The findings from the current study showed slightly greater variability (81% with either one line or no difference on retest). This may be due to the smaller sample size or because vision loss was simulated rather than testing people with an existing vision problem. However, reports of test-retest variability using the ETDRS chart show considerable variation, with studies defining a change needing to be greater than one (Rosser et al. 2003) or two (Rosser et al. 2001) lines difference to identify a significant change. Results from this study agree as there was a maximum of two participants (6%) above two lines difference on any test.

Sources of potential variability were controlled where possible by keeping conditions standardised and limiting the number of testers, but the use of Bangerter filters introduced a degree of variability. It is known that the VA measured through the filters does not correspond well with their labelled density designation (Odell et al. 2008; Perez et al. 2010), but this was controlled for by implementing a screening phase to select a filter that ensured the VA through the filter was within the testing range. During testing, subjects reported that the filters were not homogenous, with vision varying depending on where they were looking through the filter. To minimise this impact, subjects were monitored and asked to keep their head still when movement was seen and to hold the filter against their face to minimise any movement. While the impact was minimised, there was a degree of variability attributable to the use of these filters.

The decision to compare the Amblyopia Tracker app to the iSight app was to eliminate any impact of the presentation of the optotype on acuity, as slight (but not statistically significant) differences have been reported between clinical charts and VA tests presented on digital devices (Ansell et al. 2020; Bastawrous et al. 2015). The ETDRS chart is considered to be the gold standard for clinical care, with studies typically comparing the results to this test. However, the decision was made to compare to another app (using ETDRS letters) as the app is designed to be a standalone device utilised to detect change, where the aim of this study was to determine if the method of changing distance rather than optotype size was valid. Therefore, rather than adding another test and introducing potential fatigue effects, comparison with the ETDRS test was not included.

This initial phase provides a proof of concept, but the aim of the app is for it to be used by parents/guardians in the home environment to allow them to track changes in VA. The app tutorial does explain the need to ensure test distances are accurate, but it is anticipated that children would be more likely to move during testing than the adults in this study, increasing the variability in test distance. When testing VA at 40 cm, a reduction of 8 cm would be required to improve vision by one line, but with increasing test distances much larger movements would need to be made to result in an impact on VA (Tidbury & O’Connor 2015). At 40 cm, the fixed size optotype on the Amblyopia Tracker app is approximately equal to a VA of 0.9 logMAR, so additional caution may be required when testing children with VA at this level.

The data presented here are from the first phase evaluating the Amblyopia Tracker app. This is an important step given that assessment at home introduces more sources of variability. During the COVID-19 pandemic, the use of apps to assess VA at home was evaluated, but one study had a very low participation rate (only 16% of parents that gave consent to participate provided data) (Painter et al. 2021), and another reported a modest correlation with clinical tests (Silverstein et al. 2021). Therefore, further evaluation of the Amblyopia Tracker app is underway, with parents testing children at home and then testing repeated on return to the clinic. Parental views, via an online questionnaire, are being sought in relation to usability in addition to measuring the test-retest variability.

## Conclusion

Changing the method of measuring the threshold VA from altering optotype size to changing the test distance produces comparable results. Results are comparable between the Amblyopia Tracker app and the iSight app, with no difference in monocular VA’s or the interocular acuity difference and similar test-retest variability. The new Amblyopia Tracker app is designed to provide a simplified method of monitoring vision between hospital visits, so further evaluation is currently in progress to address this.

## References

[1] American Academy of Ophthalmology. 2020. Using a Home Vision Test to Order Glasses Online. Available at https://www.aao.org/eye-health/tips-prevention/self-home-vision-test-order-glasses-online [Last accessed 12 November 2020].

[2] Ansell, K, Maconachie, G and Bjerre, A. 2020. Does the EyeChart App for iPhones Give Comparable Measurements to Traditional Visual Acuity Charts? Br Ir Orthopt J, 16(1): 19–24. DOI: 10.22599/bioj.146 32999989 PMC7510399

[3] Aslam, TM, Tahir, HJ, Parry, NR, Murray, IJ, Kwak, K, Heyes, R, Salleh, MM, Czanner, G and Ashworth, J. 2016. Automated Measurement of Visual Acuity in Pediatric Ophthalmic Patients Using Principles of Game Design and Tablet Computers. Am J Ophthalmol, 170: 223–227. DOI: 10.1016/j.ajo.2016.08.013 27544479

[4] Bastawrous, A, Rono, HK, Livingstone, IA, Weiss, HA, Jordan, S, Kuper, H and Burton, MJ. 2015. Development and Validation of a Smartphone-Based Visual Acuity Test (Peek Acuity) for Clinical Practice and Community-Based Fieldwork. JAMA Ophthalmol, 13(8): 930–7. DOI: 10.1001/jamaophthalmol.2015.1468 PMC5321502 26022921

[5] Chen, SI, Chandna, A, Norcia, AM, Pettet, M and Stone, D. 2006. The repeatability of best corrected acuity in normal and amblyopic children 4 to 12 years of age. Invest Ophthalmol Vis Sci, 47(2): 614–9. DOI: 10.1167/iovs.05-0610 16431958

[6] Di Foggia, E, Stoll, N, Meunier, H, Rimele, A, Ance, P, Moreau, PH, Speeg-Schatz, C and Sauer, A. 2020. A new visual acuity test on touchpad for vision screening in children. Int J Ophthalmol, 13(9): 1436–1442. DOI: 10.18240/ijo.2020.09.16 32953584 PMC7459220

[7] Dong, LM, Hawkins, BS and Marsh, MJ. 2002. Consistency between visual acuity scores obtained at different test distances: Theory vs observations in multiple studies. Arch Ophthalmol, 120(11): 1523–33. DOI: 10.1001/archopht.120.11.1523 12427067

[8] Fu, Z, Hong, H, Su, Z, Lou, B, Pan, CW and Liu, H. 2020. Global prevalence of amblyopia and disease burden projections through 2040: A systematic review and meta-analysis. Br J Ophthalmol, 104(8): 1164–1170. DOI: 10.1136/bjophthalmol-2019-314759 31704700

[9] Geddes, M, McMonnies, C and Woodward, P. 1966. The variation of visual acuity with observation distance. Australian Journal of Optometry, 49(6): 164–169. DOI: 10.1111/j.1444-0938.1966.tb04527.x

[10] Hashemi, HM, Pakzad, RM, Yekta, AP, Bostamzad, PM, Aghamirsalim, MM, Sardari, SM, Valadkhan, MM, Pakbin, MM, Heydarian, SP and Khabazkhoob, MP. 2018. Global and regional estimates of prevalence of amblyopia: A systematic review and meta-analysis. Strabismus, 26(4): 1–16. DOI: 10.1080/09273972.2018.1500618 30059649

[11] Holmes, JM, Beck, RW, Repka, MX, Leske, DA, Kraker, RT, Blair, RC, Moke, PS, Birch, EE, Saunders, RA, Hertle, RW, Quinn, GE, Simons, KA and Miller, JM. 2001. The amblyopia treatment study visual acuity testing protocol. Arch Ophthalmol, 119(9): 1345–53. DOI: 10.1001/archopht.119.9.1345 11545641

[12] Nagra, M, Vianya-Estopa, M and Wolffsohn, JS. 2020. Could telehealth help eye care practitioners adapt contact lens services during the COVID-19 pandemic? Cont Lens Anterior Eye, 43(3): 204–207. DOI: 10.1016/j.clae.2020.04.002 32336578 PMC7165279

[13] O’Connor, AR and Milling, A. 2020. Normative data for the redesigned Kay Pictures visual acuity test. J AAPOS, 24(4): 242–244. DOI: 10.1016/j.jaapos.2020.05.003 32652123

[14] Odell, NV, Leske, DA, Hatt, SR, Adams, WE and Holmes, JM. 2008. The effect of Bangerter filters on optotype acuity, Vernier acuity, and contrast sensitivity. J AAPOS, 12(6): 555–9. DOI: 10.1016/j.jaapos.2008.04.012 18706841 PMC3258514

[15] Painter, S, Ramm, L, Wadlow, L, O’Connor, M and Sond, B. 2021. Parental Home Vision Testing of Children During Covid-19 Pandemic. British and Irish Orthoptic Journal, 17(1): 13–19. DOI: 10.22599/bioj.157 PMC8269789 34278213

[16] Perez, GM, Archer, SM and Artal, P. 2010. Optical characterization of Bangerter foils. Invest Ophthalmol Vis Sci, 51(1): 609–13. DOI: 10.1167/iovs.09-3726 19643962

[17] Rosser, DA, Cousens, SN, Murdoch, IE, Fitzke, FW and Laidlaw, DA. 2003. How sensitive to clinical change are ETDRS logMAR visual acuity measurements? Invest Ophthalmol Vis Sci, 44(8): 3278–81. DOI: 10.1167/iovs.02-1100 12882770

[18] Rosser, DA, Laidlaw, DA and Murdoch, IE. 2001. The development of a “reduced logMAR” visual acuity chart for use in routine clinical practice. Br J Ophthalmol, 85(4): 432–6. DOI: 10.1136/bjo.85.4.432 11264133 PMC1723918

[19] Rowe, F, Hepworth, L, Howard, C and Lane, S. 2020. Orthoptic Services in the UK and Ireland During the COVID-19 Pandemic. Br Ir Orthopt J, 16(1): 29–37. DOI: 10.22599/bioj.153 32999991 PMC7510392

[20] Royal College of Ophthalmologists. 2020. Resumption of Paediatric Ophthalmology Services During COVID-19. Available at https://www.rcophth.ac.uk/wp-content/uploads/2020/11/Resumption-Of-Paediatric-Ophthalmology-Services-During-COVID-19.pdf [Last accessed 12 November 2020].

[21] Silverstein, E, Williams, JS, Brown, JR, Bylykbashi, E and Stinnett, SS. 2021. Teleophthalmology: Evaluation of Phone-based Visual Acuity in a Pediatric Population. Am J Ophthalmol, 221: 199–206. DOI: 10.1016/j.ajo.2020.08.007 32791067 PMC7417903

[22] Steren, BJ, Young, B and Chow, J. 2021. Visual Acuity Testing for Telehealth Using Mobile Applications. JAMA Ophthalmol. DOI: 10.1001/jamaophthalmol.2020.6177 PMC7809609 33443550

[23] Tidbury, LP and O’Connor, AR. 2015. Testing vision testing: Quantifying the effect of movement on visual acuity measurement. Eye (Lond), 29(1): 129–35. DOI: 10.1038/eye.2014.247 25341437 PMC4289837

[24] Vagge, A and Nelson, LB. 2017. Compliance with the prescribed occlusion treatment for amblyopia. Curr Opin Ophthalmol, 28(5): 454–459. DOI: 10.1097/ICU.0000000000000409 28617688

[25] Zhao, L, Stinnett, SS and Prakalapakorn, SG. 2019. Visual Acuity Assessment and Vision Screening Using a Novel Smartphone Application. J Pediatr, 213: 203–210.e1. DOI: 10.1016/j.jpeds.2019.06.021 31326117

